# Moderate Alcohol Intake in Non-Alcoholic Fatty Liver Disease: To Drink or Not to Drink?

**DOI:** 10.3390/nu11123048

**Published:** 2019-12-13

**Authors:** Maria L. Petroni, Lucia Brodosi, Francesca Marchignoli, Alessandra Musio, Giulio Marchesini

**Affiliations:** Department of Medical and Surgical Sciences, “Alma Mater” University, Sant’Orsola-Malpighi Hospital, Via Massarenti 9, 1-40135 Bologna, Italy; luciabrodosi@gmail.com (L.B.); francesca.marchignoli@gmail.com (F.M.); alessandramusio87@gmail.com (A.M.)

**Keywords:** drinking pattern, safe alcohol intake, cardiovascular system, liver disease

## Abstract

Nonalcoholic fatty liver disease (NAFLD) is defined by hepatic steatosis in the presence of alcohol intake within safe limits, defined by guidelines of scientific associations (usually 20 g or 2 units/day in women, 30 g or 3 units in men). The diagnosis is usually followed by medical counseling of total abstinence, in order to prevent disease progression. This policy has been challenged by epidemiological studies, suggesting that the risk of liver disease and disease progression is lower in modest drinkers than in total abstainers. We revised the literature on the effects of modest alcohol intake on disease burden. Epidemiological data may suffer from several potential biases (recall bias for retrospective analyses, difficulties in the calculation of g/day), limiting their validity. Prospective data suggest that NAFLD patients with regular alcohol intake, although within the safe thresholds, are at higher risk of liver disease progression, including hepatocellular carcinoma; a detrimental effect of modest alcohol drinking is similarly observed in liver disease of viral etiology. Alcohol intake is also a risk factor for extrahepatic cancers, particularly breast, oral, and pharyngeal cancers, with gender difference and no floor effect, which outweigh the possible beneficial effects on cardiovascular system, also derived from retrospective studies. Finally, the negative effects of the calorie content of alcohol on dietary restriction and weight loss, the pivotal intervention to reduce NAFLD burden, should be considered. In summary, the policy of counseling NAFLD patients for alcohol abstinence should be maintained.

## 1. Introduction

Nonalcoholic fatty liver disease (NAFLD) is a chronic condition characterized by the accumulation of fat within hepatocytes (steatosis), potentially progressive to nonalcoholic steatohepatitis (NASH), where hepatic necroinflammation and fibrosis add to steatosis, favoring progression to cirrhosis and terminal liver failure. The term “nonalcoholic” was coined to differentiate the disease from similar histologic findings present in subjects who abuse of alcohol (alcoholic fatty liver disease (AFLD)). The difference is based on the amount of alcohol intake. By definition, individuals with NAFLD are expected to consume alcohol in amounts considered not at risk of hepatic involvement [1]. This definition raises the important question of the “safe” amount of alcohol consumption, considering that a moderate alcohol intake is very common in the population, specifically during familial or social events (social drinkers), expectedly at rates not associated with any untoward effects and possibly with positive effects on cardiovascular disease [2]. Large epidemiological data report a J-shaped curve between mortality and alcohol intake [3], with a reverse point (the point where mortality in abstainers equals mortality in alcohol users) for an average alcohol consumption of approximately 25–30 g in women and 40 g in men [4].

All NAFLD studies explicitly report the exclusion of subjects with alcohol intake at risk, defined according to pre-specified criteria, and most clinicians suggest total abstinence from alcohol as therapeutic measure for prevention NAFLD and/or disease progression. More than a decade ago a few epidemiological studies reported an inverse association between moderate alcohol consumption (within presumably safe limits) and the prevalence of NAFLD in the population [5,6], pointing to a favorable effect of moderate alcohol use extending from the cardiovascular system to the setting of metabolic liver disease. This raised a lot of debate on the correct dietary and lifestyle treatment of NAFLD, which has not settled yet. Do we need to counsel our NAFLD patients for total alcohol abstinence to prevent disease progression?

This narrative review is intended to summarize the evidence linking moderate alcohol intake with NAFLD, starting from difficulties in the correct assessment of alcohol intake. The conclusions are based on the experience of authors and the general effects of alcohol on the liver and outside the liver, in the hope to generate a common health policy among healthcare professionals.

## 2. Literature Search

A literature search was carried out on PubMed using the index terms “NAFLD” or “nonalcoholic fatty liver” and “alcohol intake” for the period 1 January, 2000–30 September, 2019. Studies were included if: (1) they were observational studies aimed at measuring the effects of modest drinking and the dose response between alcohol use and liver disease; (2) NAFLD was defined by any test, liver enzymes, surrogate measures of liver disease, imaging, and eventually histology. This search produced 334 references, including several review articles that were analyzed by authors. Further manual searching for additional articles was done using relevant references of selected articles. Finally, we identified 15 original studies (Table 1) and three review articles [7,8,9] which are the basis of the present report.

## 3. Assessment of Alcohol Intake

### 3.1. Use or Abuse?

Current guidelines on NAFLD strictly indicate the limits of alcohol intake to dissect NAFLD and AFLD; an accurate screening for alcohol intake is mandatory for a correct diagnosis, but very few studies identify the tool(s) used for alcohol assessment. In most cases this piece of information is derived from patients themselves, and confirmed by relatives in a few cases, or simply derived from brief enquiry on family use.

A correct assessment of alcohol intake should involve a wider use of questionnaires; they were specifically developed for detecting alcohol abuse, not to grade modest/moderate alcohol intake. The 4-item CAGE (acronym for the initial letters of the four questions) questionnaire is a very short screening test for the diagnosis of lifetime alcohol abuse/dependence [10], but very rarely the four questions are all used to screen patients correctly. The questionnaire is probably totally insensitive to recent alcohol drinking [11], and scarcely applies to modest (social) drinkers. The Alcohol Use Disorder Identification Test (AUDIT, 10 items) is better than CAGE to identify subjects with recent alcohol use or dependence [12], but, as far as we know, rarely used to interview patients in busy liver units considering the length of enquiry. A shorter version of AUDIT (AUDIT-C), consisting of only three questions, has been developed and more largely used, with good specificity for heavy drinking and dependence [13], but its effectiveness to diagnose moderate alcohol drinking has never been extensively investigated. The 3 questions of AUDIT-C (*1. How often do you have a drink containing alcohol? 2. How many drinks containing alcohol do you have on a typical day when you are drinking? 3. How often do you have six or more drinks on one occasion?*) have been further simplified suggesting that question #1 might suffice, provided that a correct identification of the frequency of alcoholic drinks is carried out. Another single-question screening tool has been validated for identifying individuals consuming alcohol at risky levels in primary care: “*How many times in the past year have you had 5 (for men; 4 for women) or more drinks in a day?*” [14]. If the answer is one or more, further assessment is mandatory.

### 3.2. How much Alcohol Is in a Single Drink?

The amount is likely to make the difference. In order to facilitate comparison between different studies, there has been a progressive agreement to focus on multiple of 10 g per single alcohol unit (1 glass of wine, 1 can of beer, a shot of hard alcohol drink). The harmonization of the amounts defined in AUDIT questions becomes mandatory in order to have the safe amount of alcohol correctly defined.

Following a rather long period where amounts and/or units were differently calculated in the various Countries, there is now agreement that the safe limits of alcohol use may be limited to 20 g/day in females and 30 g/day in males, with one unit corresponding to 10 g intake. These thresholds have been accepted by American and European guidelines [15,16], with minimal but significant differences: 2 and 3 units/day in Europe, 14 and 21 units/week in the United States for women and men, respectively. This amount does not consider the detrimental effect of binge alcohol drinking, particularly in the young, and the debated cumulative effect of alcohol intake along the years in the elderly [17]. Lifetime alcohol intake is based on recall over long periods; it might be relatively easy to calculate in heavy drinkers, consuming alcohol regularly at meals, but may become a very difficult task in social drinkers, consuming alcohol only during special events.

In summary, the definition of alcohol intake as assessed by questionnaires still reflects a compelling need to identify alcohol abuse and the risk of alcoholic cirrhosis. The identification of modest alcohol intake, although in excess of safe limits, and the computation of lifetime alcohol intake remain at best imprecise.

## 4. Moderate Alcohol Consumption on NAFLD: The Evidence for Protection 

The evidence for a positive effect of moderate alcohol intake on fatty liver are reported in Table 1 [5,6,18,19,20,21,22,23,24,25,26,27,28,29,30]. Rumors date back to 2001, when Dixon et al. reported an analysis of alcohol consumption in 105 patients whose liver disease was diagnosed by laparoscopic biopsies taken at surgery for severe obesity (BMI > 35 kg/m^2^) [18]. NASH was present in 26 cases (11 with advanced fibrosis). In the whole cohort, moderate alcohol consumption was associated with a decreased risk of NASH (OR, 0.35; 95% CI, 0.12–1.00). The authors suggested a possible beneficial role of alcohol mediated by reduced insulin resistance, since the effects of alcohol on NASH were no longer significant after controlling for insulin resistance.

In a seminal study in a general population, Suzuki et al. analyzed the association between alcohol consumption (none or minimal, <70 g/week; light, ≥70 and <140 g/week; moderate, ≥140 and <280 g/week; excessive, ≥ 280 g/week) and elevated aminotransferase levels in male subjects (age 20–59 years old; mean BMI, 23.4 kg/m^2^) without any form of chronic liver diseases, undergoing annual check-up [5]. As expected, excessive alcohol consumption (≥280 g/week) was associated with raised aminotransferase levels (27.0% vs. 17.4% in subjects with none or minimal intake), whereas light and moderate alcohol intake were associated with a reduced prevalence of altered liver enzymes in the older and younger age group, respectively. During a 5-year follow-up of 326 subjects without fatty liver or altered liver enzymes at baseline, moderate alcohol consumption was associated with decreased incidence of raised liver enzymes, and the authors concluded for a positive effect of moderate alcohol requiring further investigation.

The above study was followed by a series of epidemiological data, largely confirming the results. In a large cohort of male participants in Third National Health and Nutrition Examination Survey who did not consume alcohol (*n* = 7211) or reported exclusive wine consumption up to 10 g/day (*n* = 945) [19], the presence of NAFLD was suspected on the basis of unexplained elevation of alanine aminotransferase levels using both the traditional threshold of 43 U/L or the updated healthy ranges of >30 U/L for men and >19 for women [31]. Wine consumption was systematically associated with a decreased risk of elevated aminotransferase levels, whereas modest beer or liquor drinking had no positive effect. Also mixed drinking was associated with reduced risk of NAFLD (OR 0.62; 95% CI 0.41–0.92).

Several studies on NAFLD and modest drinking were carried out in the Japanese population, a group characterized by low levels of alcohol intake and frequently involved regular check-ups including either liver enzymes or ultrasonography. The association of alcohol consumption as risk factor for ultrasonographycally-detected liver fat was tested in a large series of asymptomatic male subjects (*n* = 5599), free of hepatitis viruses and or potential hepatotoxic drug use [6]. After controlling for features of the metabolic syndrome, both light (40–140 g/week) and moderate (140–280 g/week) alcohol intake were associated with a reduced risk of fatty liver. In similar cohorts, the same authors confirmed the association of moderate alcohol consumption with reduced insulin resistance, a possible link for NAFLD protection [32]. The independent, protective effect of moderate alcohol intake on NAFLD was finally, confirmed by the same group in another study of 1138 males where steatosis was measured by CT scanning [23]. The inverse association between alcohol consumption and fat content in the liver was independent of features of metabolic syndrome, physical activity, and liver enzymes. Notably, these beneficial effects were also confirmed in a longitudinal observation of a Japanese cohort, where moderate drinking (1 U/day) reduced the risk of incident liver fat by 38% after 6 years in men [20]. Surprisingly, a study reported that the negative association between alcohol intake and liver fat at ultrasounds was also present in subjects consuming relatively large amount of alcohol, well above the accepted safe limits (≥60 g/day), and that the frequency of consumption ≥ 21 days/month (OR 0.62; 95% CI 0.53–0.71) was more relevant than total amount [21].

The effects of genders are of particular interest. Moriya et al. reported that the amount of alcohol intake was inversely associated with the prevalence US-detected NAFLD in the Japanese population of both genders (Men: OR 0.59; 95% CI 0.52–0.68; Women: OR 0.60; 95% CI 0.45–0.80) [22]. Hamaguchi et al. confirmed a favorable effect of alcohol in 18,571 participants of both genders and in a large age-range, found that the prevalence of fatty liver at CT scanning was lower in men and women who consumed alcohol than that measured in subjects with no or minimal intake [24]. Data were confirmed in a re-appraisal of the Okayama database [22], where also changes in drinking patterns of 3777 men and 1524 women were considered [27]. During follow-up, newly developed fatty liver occurred in 13.4% of men and 7.8% of women, whereas 24.2% of men and 29.8% demonstrated NAFLD remission. In both men and women changes in drinking pattern paralleled changes in the occurrence of fatty liver, and modest alcohol intake was associated with negative odds of NAFLD. Notably, in men, NAFLD was also reduced by an alcohol intake above the safe limits of 280 g/week, after adjustment for confounders.

Cohorts with biopsy-assessed liver disease were also used to define the putative role of modest alcohol intake. A protective effect was confirmed in a limited cohort of adult participants in the NIH NASH Clinical Research Network, where a modest alcohol consumption, as well as an increased frequency of alcohol intake, was associated with negative odds of NASH, hepatocellular ballooning, and fibrosis [25]. In a different series with biopsy-assessed NAFLD and very modest alcohol intake, the severity of liver disease was negatively associated with lifetime alcohol intake and limited periods of total abstinence [26]. Lifetime non-drinkers or patients who had lifetime alcohol consumption < 24 g-years more frequently had fibrosis F3/F4 group compared to the group with F0–F2 liver disease. Similarly, Hagstrom et al. found a reduced risk of fibrosis associated with incremental lifetime alcohol intake (up to 13 U/week), but a recent alcohol intake increased the risk of fibrosis [28], also in relation drinking patterns.

The influence pattern of drinking pattern on the association of modest alcohol consumption with NAFLD has been thoroughly investigated in an Australian study, where the reduced risk of severe NAFLD (advanced fibrosis) was confirmed in subjects with modest alcohol intake (<70 g/week) [29]. However, the beneficial effects were cancelled in the presence of binge drinking, defined as the consumption of more than four standard drinks (women) or five drinks (men) in one single occasion [29].

A single study tested the association of modest alcohol intake on survival in NAFLD, using data from NHANES participants from 1988 to 2010 [30]. NAFLD was diagnosed by a biomarker (Hepatic Steatosis Index [33]) in 4568 subjects; alcohol intake was defined as the consumption in the course of the 12 months before examination. After a median follow-up of 70 months, modest alcohol intake was associated with 41% reduced mortality (hazard ratio [HR] 0.64, 95% CI 0.42–0.97) after adjustment for multiple confounders. However, drinking more than 1.5 U/day was associated with increased mortality (HR 1.45, 95% CI 1.01–2.10).

In 2014–2015 a critical review and a meta-analysis tried to summarize the available evidence on the association between alcohol intake and fatty liver. In a meta-analysis involving 43,175 individuals Sookoian et al. [9] concluded for heterogeneity of studies, but the overall odds for NAFLD associated with modest alcohol consumption were indicative of a protective effect (Fixed models: OR 0.69, 95% CI 0.65–0.73; random models: OR 0.68; 95% CI 0.58–0.81). Data did not change after adjustment for multiple confounders. Similarly, they concluded for a protective effect of modest alcohol intake on NASH in subjects with NAFLD (OR 0.50, 95% CI 0.34–0.74), without evidence of heterogeneity. Ajmera et al. [7] revised 7 observational studies (six with liver-related end-points) and concluded for a negative association between alcohol intake and the risk of advanced liver disease (NASH and fibrosis). However, the methodological score of studies was graded on average as low and the positive effects might be jeopardized by binge drinking. For this reason, they did not recommend modest alcohol intake in patients with NAFLD.

In summary, a few studies are in keeping with a beneficial effect of modest alcohol intake on NAFLD occurrence and progression. However, only four out of 15 studies are prospective (Table 1). Cross-sectional analysis defines the odds of events on the basis of the present alcohol intake, but the occurrence of NAFLD and NAFLD progression is a process requiring long-term damage. The event is definitely driven by previous alcohol history, difficult to define over long periods, particularly in subjects who do not consume alcohol regularly at meals and limiting alcohol intake during special events (social drinkers).

## 5. Possible Mechanism(s) of Modest Alcohol Protection and Potential Confounders

Several mechanisms have been suggested as the basis for the negative association between modest alcohol consumption and liver fat, assumed as putative causal-effect protective effect. Moderate alcohol use has been associated with lower insulin resistance, a feature of metabolic syndrome-associated NAFLD and NAFLD progression, as observed in several epidemiological studies [34,35], including the ones reported in this review [18,32], also affecting triglyceride levels. Modest alcohol use was associated with improved lipid profile and anti-inflammatory properties, also producing a positive effect on cardiovascular system [36,37,38].

Several other factors might be involved, limiting the significance of the association. Moderate users might belong to higher socio-economic classes, with higher levels of education, more prone to physical activity, always used to consume alcohol within safe limits. On the contrary, cohorts of totally abstinent individuals might be enriched with formerly heavy drinkers, the so-called sick-sitter cohort [3], not identified in cross-sectional analyses, with significant liver disease [39], as well as with subjects with higher prevalence of obesity and other comorbid conditions. These factors are likely to produce a selection bias and reverse causality. Alternatively, the effect might be due to genetic factors specifically associated with ethnic groups, although the variety of cohorts tested in the different studies should reduce this bias.

Finally, the specific effect indicated for wine, not for beer or liquor drinking, might point to specific metabolic activity of individual constituents of alcoholic beverages. Both alcohol and non-alcohol components of wine might be involved. Resveratrol is a potential candidate, considering that it reduced or attenuated steatosis in experimental animals [40,41], although a direct effect in humans has never been definitely proven. In a randomized study vs. total abstention, moderate wine intake (3.0 and 1.5 U/day in men and women, respectively) for 3 months produced only a minimal increase in hepatic triglyceride content, insufficient to define hepatic steatosis [42]. This is the only trial on this topic, and will probably remain an isolated experiment, due to ethical concern. Wine is a component of the Mediterranean diet; totally abstinent individuals are considered non-adherent to the Mediterranean diet style as much as heavy drinkers, and the Mediterranean diet has been associated with better NAFLD outcome [43,44,45]. This made the Mediterranean diet the meal composition of choice for people with NAFLD [46], as also recommended by International guidelines [15]. The Mediterranean diet has long been associated with reduced cardiovascular risk; a recent randomized study conducted for 18 months in 278 individuals with NAFLD confirmed the superiority of a Mediterranean-style diet vs. a low fat diet on hepatic fat content, measured by magnetic resonance imaging, also in the presence of similar weight loss [47]. However, in the presence of conflicting results [48], the role of alcohol vs. other dietary components should be better defined.

## 6. Moderate Alcohol Consumption and the Liver within and outside NAFLD: The Evidence for Detrimental Effects

The evidence for protection given by moderate alcohol intake on NAFLD has however been challenged (Table 2) [49,50,51,52,53,54,55,56,57,58,59,60,61,62,63]. In the Italian Dionysos study, a study exploring the prevalence of liver disease in the general population of two Italian towns [49], alcohol use was associated with both fatty liver incidence and remission when graded for any 20 g/day in both men and women. In 71 patients with biopsy-proven NAFLD who had a second biopsy after a mean follow-up of 13.8 years, the proportion of patients reporting at risk episodic alcohol drinking (at least once a month) was higher among those with significant fibrosis progression, defined as increase by one or more fibrosis stage or occurrence of end-stage liver disease [50]. The authors concluded that patients should be advised to refrain from heavy episodic drinking. In the Finnish Health 2000 Study, a nationally representative cohort where alcohol consumption and metabolic factors were extensively investigated, alcohol was selected as risk factor for liver disease both in heavy alcohol users and in those who consumed alcohol within expected safe limits (<2–3 U/day in women and men, respectively) [51]. In a larger reappraisal of two database (FINNRISK 1992–2012 or Health 2000) comprising a selected cohort of 8345 individuals with hepatic steatosis defined by the Fatty Liver Index [64], alcohol was selected as risk factor for liver related events throughout the five strata of alcohol intake from none to 50 g/day [52]. All these subjects were at higher risk of composite fatal and non-fatal liver-related events (liver-related mortality, hospital admission, liver cancer) (hazard ratios between 2.18 and 8.79 for the different strata of alcohol intake), with a possible beneficial effect on cardiovascular outcomes.

The risks for the liver associated with alcohol use/abuse extends outside the specific setting of NAFLD and have been extensively reported in the literature since 1957 [65]. Both the daily pattern of alcohol intake, extremely variable across countries [66], and the cumulative dose of alcohol seem to produce harm to the liver [17]. In the Danish Cancer, Diet, and Health study (1993–2011), involving 55,917 participants (aged 50–64 years old), the risk for alcoholic cirrhosis among daily drinkers was 3.65 (95% CI 2.39–5.55) compared to subjects drinking only 2–4 days/week [53]. However, an individual susceptibility to the detrimental effect of alcohol for the liver may mediate the dose-response curve of progressive damage [67].

The safe limits of alcohol intake to reduce the risk of future liver disease are derived from a study on 13,285 men and women aged 30 to 79 years old, where alcohol intake was measured by a self-administered questionnaire and liver disease was derived from death certificates and hospital discharge registers [54]. The risk remained low at an alcohol intake of one to six U/week and increased in a dose-response fashion above this amount, independently of age. The increase reached statistical significance at 7–13 U/week for women and 14–27 U/week for men. Above these limits, alcohol becomes a risk factor for alcoholic cirrhosis and may be a cofactor for liver disease of other etiologies. In the Dionysos Study, a cohort study measuring the prevalence of chronic liver disease in the general population of two northern Italian communities, the risk threshold for developing either cirrhosis or non-cirrhotic liver damage was estimated at >30 g/day in both sexes [55]. The prevalence of steatosis was increased in heavy drinkers (46.4%) and in subjects with obesity (75.8%), but heavy drinkers with obesity had an even higher prevalence (94.5%), suggesting an additive effect, with obesity doubling the risk for steatosis in heavy drinkers [56]. In the same cohort, characterized by a 2.3% prevalence of HCV infection, alcohol consumption >30 g/day increased the risk of disease progression during a 3-year follow-up [57].

The synergistic effect of alcohol intake and obesity on liver disease development and progression was also demonstrated in two of the long-term Midspan prospective cohort studies in Scotland, where participants were divided in 3 by 3 groups on the basis of alcohol use (none, 1–14 U/week, ≥15 U/week) and body mass index (BMI, normalweight, overweight, obesity) [58]. Both alcohol intake and BMI were strongly associated with long-term mortality. After a median follow-up of 29 years, the rates of liver disease for drinkers of ≥15 U/week were significantly higher than in non-drinkers, irrespective of BMI status. In the cohort who consumed 1–14 U/week, an excess of liver disease was only observed in subjects with obesity, with a synergistic effect between alcohol and BMI.

The same pathogenic involvement of alcohol as cofactor for diseases holds true in patients with hepatitis virus infection. Subjects with hepatitis C virus infection report a significant alcohol intake [68], and are more prone to alcohol abuse than the general population [69], and alcohol is expected to impact on progression of chronic hepatitis [70]. Hezode et al. initially reported a progressive increase in histologic severity and fibrosis in relation to the amount of alcohol intake in chronic HCV infection [59,70]. However, in a similar cohort of 857 patients, the degree of fibrosis was independent of mild-moderate alcohol consumption [71]. A similar negative effect is expected to occur with hepatitis B infection, but also in this condition conflicting data have been reported [72], as well as in storage defect disease [73].

Certainly, even modest alcohol intake may be a cofactor for the development of hepatocellular carcinoma in non-alcohol-related liver disease. In half-million Korean participants in regular check-ups, the incidence of hepatocellular carcinoma was associated with hepatitis B and C infection, but the risk increased for any 20 g/day of alcohol consumption, particularly in the elderly [60]. A similar detrimental effect was demonstrated in NAFLD, where alcohol intake systematically increased the risk of hepatocellular carcinoma, synergistically with obesity, also in subjects with moderate alcohol consumption [8]. Notably, total abstinence prevents disease progression and improves outcomes also in subjects with liver disease of alcoholic origin [74].

The very recent analysis of the participants in FINNRISK 1992–2012 and Health 2000 database, testing the association of alcohol intake on hospital admissions, cancers and mortality, after exclusion of abstainers and heavy drinkers (>50 g/day) [52], confirmed a linear association between alcohol intake and liver malignancies, but a dose-dependent negative association with cardiovascular outcomes, with risk reduction of 21% at very modest alcohol intake (≤1 U/day) limited to non-smokers. This translated into a J-shaped curve between alcohol and total mortality, that was higher than in abstainers at alcohol use of 3 U/day. This reinforced a target organ difference for the effects of alcohol on human body.

Considering the dramatic increase in the prevalence of obesity, diabetes and metabolic syndrome, all conditions associated with potentially progressive liver disease, it is very likely that the safe limits of alcohol consumption should be reduced to avoid synergistic effects on liver disease development and progression in the general population [8]. in high-income countries, the threshold for lowest risk of all-cause mortality was set at about 100 g/week, i.e., lower than those recommended in most current guidelines [75]. The association between alcohol and obesity should also consider the contribution of alcohol to excess calorie intake (7 kcal/g). Thus, also a modest alcohol consumption may provide 100–300 kcal/day, thus explaining the association with obesity in both men and women [76,77], independent of the alcohol source [78]. This also explain why NAFLD and ALD frequently result in a similar phenotype [79] and why both may increase the risk of diabetes [80].

In summary, the evidence for supporting a detrimental effect of alcohol intake on NAFLD severity and disease progression (Table 2) is largely based on solid longitudinal studies. Excluding the studies specifically dealing modest alcohol intake as cofactor in the presence of liver disease of viral etiology [57,59,61], the association of alcohol with long-term fibrosis progression and/or cancer development seems very solid and less biased by the multiple drawbacks registered in cross-sectional analyses.

## 7. Effects of Alcohol Intake Beyond the Liver

The effects of alcohol extend well beyond the liver; the literature has been extensively revised in a Consensus document of a team of Italian experts, coordinated by the Nutrition Foundation of Italy and endorsed by several scientific Societies [81]. Two areas deserve particular attention for the interaction between alcohol and extra-hepatic disease: (a) cardiovascular disease; (b) cancer. For all areas, a relevant effect of gender was reported, with women at much higher risk than men.

### 7.1. Cardiovascular Disease

Several meta-analyses tested the association between alcohol and coronary artery disease, showing the J-shaped curve reported for total mortality, particularly in women, whereas in men there was a linear decrease in cardiovascular events for levels of alcohol consumption much higher than safe limits [82]. The effects were in the order of magnitude of a 25–40% reduction in fatal and non-fatal coronary events for an alcohol intake in the modest amounts (1–2 U/day), irrespective of the presence of diabetes or pre-existing coronary disease [81], the type of alcoholic beverage and the consumption with/without meals [36]. The risk of arrhythmias, particularly atrial fibrillation, associated with alcohol use even in moderate amounts should however be mentioned [83], as well as the risk associated with binge drinking [84].

The J-shaped curve was confirmed for stroke [85], where the consumption of alcohol above 5 U/day started to increase the risk of events, particularly in women, confirming the gender effect also reported for coronary disease [82]. Protection by modest alcohol intake added to the protection exerted by healthy lifestyles [86] and to secondary prevention in 1320 participants in the Physician’s Health Study [87]. The association with peripheral artery disease has been less clearly investigated, but the overall studies are in line with those reported above, possibly mediated by blood pressure, also showing a J-shaper curve in relation with alcohol intake. In this case the nadir was initially observed at very modest amount of alcohol (1–2 U/day) [88], with conflicting results in relation to gender [81]. Notably, the protection exerted by alcohol might also extend to subjects with established fatty liver [52]. Both the prevalence of carotid plaques and carotid artery stenosis were also reduced in subjects consuming modest amounts of alcohol (<20 g/day), and were negatively associated with alcohol use [38]. Reduced blood pressure and prevention of cerebro-vascular involvement might also account for the association of modest alcohol use with reduced incidence of cognitive dysfunction and risk of dementia reported in two meta-analyses [89,90], with a nadir at 0.5 U/day and increased risk above 3 U/day [90].

### 7.2. Cancer

The harmful effects of alcohol consumption on cancer risk have been extensively investigated; on caner outcomes, the effects of moderate alcohol intake in specific organs are beyond dispute, although the mechanism(s) have not been completely understood. The most widely tested association is the risk of breast cancers, where several studies and meta-analyses/review articles are available [91,92]. The risk, possibly mediated by the effect of alcohol on estrogen concentration, increases by 7–10% for any unit of alcohol/day, without a floor effect [92]. In the prospective observational study of 105,986 women enrolled in the Nurses’ Health Study followed and 2.4 million person-years of follow-up, also very modest alcohol consumption was associated with increased breast cancer risk, starting at levels equivalent to only 3–6 U/week, without any relation with drinking pattern, total consumption, and age [93]. A detrimental effect of modest alcohol consumption was also found for oral and pharyngeal cancer risk, whereas the effects of low dose alcohol intake on the risk of colo-rectal cancer remain uncertain and probably null. Overall, the burden of cancer associated with alcohol is extremely high; Europe is the area of heaviest consumption and higher cancer risk, with alcohol drinking accounting for an estimate 4.2% of all disability-adjusted life-years and 5.2% of deaths [94]. Apparently, no safe limits of alcohol intake may be defined to prevent cancer risk.

## 8. Conclusions

What should we recommend to our next patient with NAFLD regarding drinking habits? A few observational studies have reported beneficial effects on cardiovascular outcomes of low-to-moderate alcohol consumption in the general population as well as in patients with NAFLD [7,52]. Cardiovascular disease remains the most common cause of mortality in NAFLD/NASH [95], and any intervention aimed at reducing cardiovascular events would be a very important target of treatment. A very modest alcohol intake, possibly even lower than the diagnostic cut-offs of NAFLD, is not associated with advanced fibrosis in epidemiological studies, and might be even protective. However, data are far from solid. The protective effects are largely derived from retrospective analyses, all based on self-assessed methods and subject to several selection biases. In prospective analyses even modest alcohol intake has been associated with possible disease progression [51] and, more significantly, with cancer development [8]. In agreement with several colleagues [96,97,98], we believe that abstinence should be the goal, particularly in women, in whom the general risk for extra-hepatic cancer significantly adds to the risk for hepatocellular carcinoma, without any safe threshold. This policy is shared by International Guidelines and should be reinforced following the recent publication of the combined analysis of data from three large-scale data sources in 19 high-income countries (the Emerging Risk Factors Collaboration, EPIC-CVD, and the UK Biobank) [75]. This report found an increase in total mortality and disease-specific events rising for alcohol intake above the threshold of 100 g/week, much lower than the accepted safe limits, supporting the concept that safe limits should be corrected [75]. Sheron and Williams suggest that better drinking advice is the most effective approach for improving health [98]. Drinking pattern is part of our social life, and safe drinking should be supported by healthcare professionals as well as Government Institutions also in healthy individuals. In patients with NAFLD, as well as in the presence of liver disease of any etiology, the lower the better and the policy of counseling alcohol abstinence by healthcare professionals should be maintained.

## Figures and Tables

**Table 1:** Summary of studies suggesting a protective effect of moderate alcohol intake on NAFLD prevalence and/or NAFLD progression.

Author, Year	Type of Study/Patients	Alcohol Assessment	Outcome Measures	Results
Suzuki, 2007 [5]	Cross sectional and prospective community-based study. 1177 male subjects with annual check-up. 326 subjects without elevated ALT with had a 5-year F-UP	Questionnaire. Alcohol graded as none, light, moderate, excessive	Raised ALT	Light (70–140 g/week: OR 0.6; 95% CI 0.4–1.0) and moderate (140–208 g/week: OR 0.5; 95% CI 0.3–0.9) alcohol consumption was negatively associated with raised ALT in the older and younger groups, respectively, vs. subjects with none or minimal intake. At F-UP, moderate intake was associated with decreased incidence of raised ALT (adjusted HR 0.4; 95% CI 0.1–0.9)
Gunji, 2009 [6]	Cross-sectional, community-based study. 5599 Japanese men with regular medical survey	Questionnaire. Alcohol graded in g/week	US-detected fatty liver	Both light (40–140 g/week) and moderate (140–280 g/week) alcohol intake independently reduced the risk of fatty liver (OR 0.82; 95% CI 0.68–0.99 and OR 0.75; 0.61–0.93)
Dixon, 2001 [18]	Cross sectional cohort study. 105 patients with liver disease submitted to bariatric surgery	Medical consultation, questionnaire	Presence of biopsy-proven NASH	Moderate alcohol consumption was associated with a decreased risk of NASH (OR, 0.35; 95% CI, 0.12–1.00).
Dunn, 2008 [19]	Cross sectional, community-based study of 7211 NHANES III participants not consuming alcohol and 945 wine drinkers	Questionnaire. Modest consumption: defined <100 g/week	Raised ALT (both laboratory range and updated definition) [31]	Irrespective of the reference cut-point, modest wine consumption was associated with 50% reduced risk of elevated ALT (OR 0.51; 95% CI 0.33–0.79 with updated normal ranges); no effect was demonstrated for beer or liquor drinking, whereas mixed drinking was protective.
Gunji, 2009 [6]	Cross-sectional, community-based study. 5599 Japanese men with regular medical survey	Questionnaire. Alcohol graded in g/week	US-detected fatty liver	Both light (40–140 g/week) and moderate (140–280 g/week) alcohol intake independently reduced the risk of fatty liver (OR 0.82; 95% CI 0.68–0.99 and OR 0.75; 0.61–0.93)
Yamada, 2010 [20]	Cross-sectional + longitudinal study (5444 men, 4980 women on regular check-ups. F-UP, 6 years	Frequency and amount of drinking in g/week	US-detected fatty liver	Occasional, daily moderate (1 U/day) or heavy (≥ 2 U/day) drinking was negatively associated with liver fat. On follow-up, moderate drinking maintained a negative association with fatty liver in men (OR 0.72, 95% CI 0.58–0.89), not in women
Hiramine, 2011 [21]	Cross-sectional cohort (9886 males on regular health check-ups)	Questionnaire. Classified as none, light (≤ 20 g/day), moderate (20–59), heavy	US-detected fatty liver	Fatty liver was positively associated with obesity and negatively alcohol intake (light, OR 0.71, 95% CI 0.59–0.86; moderate, OR 0.55, CI 0.45–0.67; heavy, OR 0.44, CI 0.32–0.62). The frequency of alcohol consumption was more relevant than total amount.
Moriya, 2011 [22]	Cross sectional, community-based study. 4957 men and 2155 women, median age 49, free of known liver disease	Lifestyle data derived from questionnaire (g/day)	US-detected NAFLD. Raised ALT by updated reference [31]	The prevalence of fatty liver was significantly lower in drinkers than in nondrinkers (28% vs. 40% in men and 10% vs. 16% in women (*p* < 0.001 for both). NAFLD prevalence was inversely associated with both amount and frequency of alcohol intake.
Gunji, 2012 [23]	Cross-sectional, community-based study. 1138 Japanese men with regular check-up, age ≥ 40	Questionnaires	CT-detected NAFLD	Alcohol consumption was associated with a reduced risk of liver fat, independently of features of metabolic syndrome, physical activity ad raised liver enzymes.
Hamaguchi, 2012 [24]	Cross-sectional community-based study. 8571 Japanese men and women, aged 18–88; mean BMI, 22.6 kg/m^2^	Questionnaires. Alcohol intake categorized into 4 grades	Fatty liver by ultrasounds	For both men and women, light and moderate alcohol intake was inversely associated with fatty liver (Men: OR 0.69, 95% CI 0.60–0.79 and OR 0.72, 95% CI 0.63–0.83; Women: OR 0.54, 95% CI 0.34–0.88 and OR 0.43,95% CI 0.21–0.88).
Dunn, 2012 [25]	Cross-sectional cohort study. 251 lifetime modest drinkers; 331 non-drinkers (NIH NASH CRN)	AUDIT test. Alcohol intake <140 g/week: extensive analysis of drinking pattern	Liver biopsy	Modest drinking within safe limits reduced the odds of NASH (OR 0.56, 95% CI 0.39–0.84), fibrosis (OR 0.56; 95% CI 0.41–0.77) and ballooning (OR 0.66, 95% CI 0.48–0.92) vs. lifetime non-drinking habits
Kwon, 2013 [26]	Cross-sectional cohort study. 77 patients with biopsy-assessed NAFLD, alcohol intake < 40 g/day	Lifetime retrospective alcohol intake by questionnaire	Liver biopsy	Increasing age (OR 1.07, 95% CI 1.01–1.14) was associated with more severe liver disease, whereas lifetime alcohol intake ≥ 24 g-years was associated with less severe disease (OR 0.26, 95% CI 0.07–0.97).
Moriya, 2015 [27]	Prospective analysis of several community-based cohorts (3773 men and 1524 women); F-UP, NS	Questionnaire	US-assessed NAFLD incidence	In both men and women, modest alcohol intake was associated with negative odds of NAFLD. In men, NAFLD was also reduced by alcohol intake in the range ≥280 g/week, after adjustment for confounders (OR 0.68; 95% CI 0.58–0.79)
Hagstrom, 2017 [28]	Cross-sectional, cohort study. 120 subjects with biopsy-proven NAFLD	Questionnaires for lifetime alcohol intake. PEth for recent alcohol	Liver biopsy	Alcohol intake up to 13 U/week was associated with reduced risk of fibrosis (OR 0.86 per U/week, 95% CI 0.76–0.97). High PTth was associated with a higher risk of fibrosis (OR 2.77, 95% CI 1.01–7.59)
Mitchell, 2018 [29]	Cross-sectional, cohort study. 187 NAFLD patients (24% with advanced fibrosis)	Questionnaires for previous and actual alcohol intake and binge drinking	Liver biopsy	Modest consumption was associated with a decreased risk of advanced fibrosis (OR 0.33, 95% CI 0.14–0.78). The association was not confirmed in binge drinking. Exclusive wine, not beer drinking, was negatively associated with advanced fibrosis (OR 0.20, 95% CI 0.06–0.69), compared to lifetime abstinence.
Hajifathalian, 2018 [30]	Prospective, community-based study of 4568 NHANES participants. F-UP, 70 months	Questionnaire for amount and type of alcohol drinking	Hepatic Steatosis Index [33]	Modest alcohol consumption was associated with decreased overall mortality (HR 0.64, 95% CI 0.42–0.97 for a drinking pattern of 0.5–1.5 U/day). However, in NAFLD alcohol consumption ≥ 1.5 U/day had a harmful effect on mortality (HR 1.45, 95% CI 1.01–2.10), after adjustment for confounders.

Abbreviations: ALT—alanine aminotransferase; AUDIT—Alcohol Use Disorder Identification Test; BMI—body mass index; CI—confidence interval; CT—computed tomography; F-UP—follow-up; HR—hazard ratio; NS—not specified; NHANES—National Health and Nutrition Examination Survey; NIH NASH CRN—National Institute of Health NASH Clinical Research Network; OR—odds ratio; PEth—phosphatidyl ethanol; US—ultrasonography.

**Table 2:** Summary of studies suggesting a detrimental effect of alcohol intake, although moderate, on the liver within and outside NAFLD.

Author, Year	Type of Study/Patients	Alcohol Assessment	Outcome Measures	Results
Bedogni, 2007 [49]	Cohort study. 144 subjects without and 336 with fatty liver (FL); F-UP, 8.5 years	Food frequency questionnaire	Fatty liver incidence and remission	For any 20 g/day increase of alcohol intake, incident FL increased by 17%, FL remission decreased by 10%, liver disease was persistent, and mortality increased by 10% in the FL cohort.
Eckstedt, 2009 [50]	Cohort study. 71 patients with biopsy-proven NAFLD; F-UP, 13.8 years	Validated questionnaire + oral interview	Significant fibrosis progression (+1 stage) or ESLD	Episodic drinking (at least 1/month) (*p* = 0.003) and higher weekly intake were more common among those with fibrosis progression. Heavy episodic drinking was an independent predictor of significant fibrosis progression.
Aberg, 2018 [51]	Cohort study. 6732 subjects without baseline liver disease; F-UP, 11.4 years	Questionnaire. Alcohol graded in g/week	Liver disease progression, HCC, liver-related death	Among subjects with non-risky alcohol intake (<3 U/day in men and 2 U/day in women), alcohol use remained as significant independent predictor of outcomes.
Aberg, 2019 [52]	8345 persons with hepatic steatosis (FLI > 60); alcohol intake < 50 g/day; F-UP, 11.1 years	Questionnaire. Alcohol graded in g/week	Imaging, liver function tests	Low alcohol intake (10–19 g/day) doubled the risk for advanced liver disease compared to lifetime abstainers. Moderate intake (up to 49 g/day) reduced the risk of incident CVD. Alcohol intake >30 g/day increased the risk for all-cause mortality compared to lifetime abstainers.
Askgaard, 2015 [53]	55,917 participants in the Danish Cancer, Diet, and Health study (1993–2011); F-UP, 14.9 years	Total alcohol intake converted into standard drinks	Clinically-detected alcoholic cirrhosis	257 and 85 incident cases of alcoholic cirrhosis were registered among men and women, respectively, none among life-time abstainers. Daily drinking increased the risk of alcoholic cirrhosis in men (HR, 3.65; 95% CI, 2.39–5.55), compared to drinking 2–4 days/week
Becker, 1996 [54]	Population-based prospective cohort study of 13,285 men and women. F-UP, 12 years	Self-administered questionnaire	Alcohol-induced liver disease by death certificates/hospital registers	The lower estimated relative risk of liver disease was observed at an alcohol intake of 1–6 U/week beverages per week. The relative risk was significantly >1 at 7–13 U/week for women and 14–27 for men.
Bellentani, 1997 [55]	Community-based study. 6917 subjects of the general population	Semiquantitative color-illustrated food questionnaire	Cirrhosis by blood tests and clinical	After the age of 50, the risk of chronic liver disease and cirrhosis was higher in those who drank alcohol. The risk increased by increasing alcohol intake above the safe limit of 30 g/day.
Bellentani, 2000 [56]	Cohort study; cross sectional analysis of 257 participants of the Dionysos Study	Validated food questionnaire	US-detected NAFLD; LFT	The risk for steatosis was 2.8-fold higher in drinkers (95% CI, 1.4–7.1), 4.6-fold higher in obese persons (95% CI, 2.5–11.0), and 5.8-fold higher (95% CI, 3.2–12.3 in persons who were obese and consumed alcohol.
Bellentani, 1999 [57]	Community-based study. 6917 subjects; 162 HCV-RNA positive. F-UP, 3 years	Validated food questionnaire	LFT, Imaging	Alcohol intake above 30 g/day 3-fold increased the risk of developing cirrhosis in HCV RNA positive drinkers. All five patients with HCC were alcohol abusers
Hart, 2010 [58]	Prospective cohort study of 9559 men in Scotland; Median F-UP, 29 years	Self-reported questionnaire	ICD-X or ICD-iX codes and NHS registries	Both BMI and alcohol consumption were associated with mortality. Higher rates of liver disease were demonstrated in consumers of ≥15 U/week of alcohol, irrespective of BMI. In modest users (1–14 U/week) liver disease rates were higher in the presence of obesity, with significant interaction (synergy index, 2.89; 95% CI, 1.29–6.47)
Hezode, 2003 [59]	Cohort study. 260 patients with chronic hepatitis C	Validated questionnaire	Liver biopsy	Histological activity and fibrosis gradually increase according to the amount of alcohol ingested, and even moderate consumption (31–50 g/day in men and 21–50 g/day in women) may aggravate histological lesions.
Yi, 2018 [60]	Cohort study. 504,646 Korean patients undergoing check-ups. F-UP, 10.5 years	Lifetime alcohol intake by questionnaire	ICD-X discharge codes	Cirrhosis increased the incidence of HCC by 42 folds, HBV by 21 folds, HCV by 19 folds, male sex by 4.3 folds. The risk of HCC increased progressively with advancing age for any 20 g/day of alcohol consumption.
Loomba, 2010 [61]	2260 Taiwanese men positive for HBV infection; F-UP, 14 years	Questionnaire for alcohol intake	HCC by imaging or histopathology (Cancer Registry)	Alcohol intake (any amount) synergistically increases the risk of incident HCC in overweight (HR, 2.4; 95% CI, 1.3–1.4), obese (HR, 2.0; 95% CI, 1.1–3.7) and extremely obese (HR, 2.9; 95% CI, 1.0–8.0) HB infected individuals
Ascha, 2010 [62]	Cohort study. Adult patients with cirrhosis secondary to chronic HCV (*n* = 315) or NASH (*n* = 195); F-UP, 3.2 years	Alcohol intake defined as never, social, significant, past	HCC by imaging or histopathology	Never drinking alcohol reduced the risk to develop HCC compared with any level of drinking (*p* < 0.001) in both the HCV and NASH groups. NAFLD patients who reported any regular alcohol intake were at greater risk for HCC development (HR, 3.6; 95% CI, 1.5–8.3).
Loomba, 2013 [63]	Population-based study. 23,712 Taiwanese subjects; F-UP, 11.6 years	Questionnaire for lifetime alcohol intake	HCC by imaging or histopathology (Cancer Registry)	Alcohol intake (any amount) and obesity synergistically increase the risk of HCC (HR, 3.82; 95% CI, 1.94–7.52) after adjustment for confounders (including diabetes and hepatitis virus infections.

Abbreviations: CI—confidence interval; ESLD—end-stage liver disease; F-UP—follow-up; FL—fatty liver; FLI—Fatty liver index; HBV—hepatitis B virus; HCC—hepatocellular carcinoma; HCV—hepatitis C virus; HR—hazard ratio; ICD—International Classification of Disease; NHS—National Health Service; US—ultrasounds.
